# Dental Department of the University of Pennsylvania-Commencement

**Published:** 1879-04

**Authors:** 


### DENTAL DEPARTMENT OF THE UNIVERSITY OF PENNSYLVANIA—COMMENCEMENT.

The first commencement of this Institution was held in the Academy of Music, March 15th, '79 at twelve m. The commencement of the Medical department was held at the same time and place. A very large audience was present.

The degree of Doctor of Dental Surgery, was conferred upon the following persons, viz:

Pennsylvania, J. Dorrance Dow, Charles G. Joy, Frank Morton Long, William H. Masser, Louis DeL. Moss; Robert J. Nickell, John Ramsden, Charles G. Walker. Minnesota, Henry C. Albright. Michigan, A. Lactance Brodeur, Iowa, Geo. C. Brown. Delaware, Henry Garrett, Jr. California, Walter LaT, Graves. Massachusetts, W. Wayland Hayden, Alfred J. Nims. New Jersey, Levi W. Johnston. Ohio, Willoughby D. Miller. Illinois, Edwin C. Timeran. Connecticut, Alfred H. Schofield, Scotland, Alexander McG. Denham, Saxony, Fritz Hroch. Ireland, Wm. J. O'Doherty. Italy, Geo. H. Van Meter. Switzerland, Adolph Wetzel. Canada, Stephen L. Wiggins.

Prof. Charles J. Essig, Secretary of the Department of Dentistry, awarded W. D. Miller, of Ohio, a set of instruments, offered by S. S. White, for the best thesis on the conservative treatment of the dental pulp; A. McG. Denham, of Scotland, the Essig prize of fifty dollars; Adolph Weitzel, of Switzerland, the prize offered by S. S. White, for the best specimen of mechanical dentistry, and A. Scofield, of Connecticut, the prize offered by Professor Darby for best specimen of gold filling.

The valedictory address was then delivered by Dr. John Ashurst, Professor of Clinical Surgery.

Thus ends the first session of this Institution, and with such a showing as to indicate a bright and successful career for the future. And may the most sanguine hope of its friends be realized.

